# Determination of Groundwater Vulnerability for Protection of the Drinking Water Basins: A Case Study From Türkiye, Kesikköprü Dam Lake Basin

**DOI:** 10.1002/gch2.202500237

**Published:** 2025-06-13

**Authors:** Olcay Gülçiçek Uysal, Kağan Cebe, Mert Cüylan, Olgu Yurttaş

**Affiliations:** ^1^ Faculty of Engineering Department of Environmental Engineering Mersin University Çiftlikköy Campus Mersin 33110 Türkiye; ^2^ Faculty of Engineering Department of Civil Engineering Ondokuz Mayıs University Samsun 55100 Türkiye; ^3^ CubicGEO Company Konutkent Mah. 3028.Cad. West Gate Residence C‐Blok No:13 Çankaya Ankara 6810 Türkiye; ^4^ Graduate School of Natural and Applied Sciences Ankara University Ankara 06560 Türkiye

**Keywords:** aquifer protection, Central Anatolia, groundwater pollution risk, vulnerability mapping

## Abstract

This study investigates the intrinsic groundwater vulnerability of the Kesikköprü Dam Lake Basin, a critical drinking water source for Ankara, Central Anatolia Türkiye. The DRASTIC model is applied within a Geographic Information Systems (GIS) framework, using seven hydrogeological parameters to generate a vulnerability map. Results indicate that most of the basin falls within “very low” and “low” vulnerability zones, with “medium” vulnerability observed in localized recharge‐prone areas. Model validation is conducted using nitrate and total organic carbon concentrations, which represent pollution from agricultural and domestic wastewater sources. The spatial correlation between these indicators and DRASTIC output supports the model's reliability. Importantly, this research also integrates anthropogenic influences by evaluating the spatial distribution of agricultural, wastewater, and mining activities relative to vulnerability zones. While natural conditions suggest low contamination potential, the cumulative and long‐term effects of these activities highlight significant risks in certain areas. To the best of the knowledge, this is the first study to validate a DRASTIC‐based vulnerability map for the Kesikköprü Basin while addressing anthropogenic pressures. The findings offer a practical decision‐making tool for land‐use planning and sustainable groundwater management, both regionally and in similar hydrogeological contexts worldwide.

## Graphical abstract

Groundwater vulnerability is evaluated in a critical drinking water basin under the threats of climate change and population growth. Using GIS‐based DRASTIC modeling and field validation, high‐risk zones are identified. The results offer strategic insights for protecting Ankara's groundwater resources against escalating environmental and anthropogenic pressures.

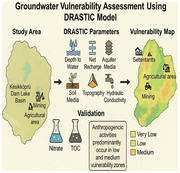

## 1 Introduction

Groundwater, a magical gift of nature, is one of the purest natural water resources essential for humanity. More than 1.5 billion people worldwide depend on groundwater for survival^[^ ^1^ ^]^ and ≈33% of the world's population uses groundwater to meet their daily needs.^[^ ^2^ ^]^ However, groundwater availability has decreased with the rising population in recent years, and water quality is deteriorating due to human activities.^[^ ^3^ ^]^ As a result, adverse changes in groundwater storage and quality worldwide have become a resource‐threatening issue.^[^ ^2^, ^3^, ^4^ ^]^ Groundwater is the main source for delivering drinking water to the world's population and, most importantly, for providing clean drinking water in developing countries and arid regions.^[^ ^5^ ^]^

In recent years, two major factors impacting the availability and quality of water resources in basins are human activities and climate change.^[^ ^6^, ^7^, ^8^, ^9^ ^]^ The careless exploitation of water resources in large basins has resulted in severe environmental issues such as soil salinization, desertification, soil cracking, and ground subsidence.^[^ ^10^, ^11^ ^]^ Sustainable management of water resources in basins around the world has thus become a critical issue, necessitating systematic and comprehensive scientific research. With the ongoing effects of climate change, industrialization, and economic growth, the global demand for water is rising. Since most surface water that could meet this increasing demand is polluted, groundwater resources are often preferred. Therefore, the development and protection of groundwater resources are essential.^[^ ^12^ ^]^

Groundwater is difficult to pollute, but once contaminated, it is challenging to rehabilitate. Although groundwater is a critical component of sustainable development, it has not received sufficient attention to protect it from pollution.^[^ ^13^ ^]^ Groundwater vulnerability refers to the probability of contaminants reaching a specific location within the groundwater system after entering the aquifer. This concept includes intrinsic sensitivity, which pertains to the characteristics that influence the migration of contaminants toward groundwater.^[^ ^14^, ^15^ ^]^ The vulnerability of groundwater to anthropogenic surface contaminants is primarily governed by the hydrogeological characteristics of the area, geological layers, soil properties, meteorological factors, and the aquifer itself.^[^ ^16^ ^]^ The interplay of these factors creates a dynamic system that determines groundwater vulnerability.^[^ ^14^ ^]^

There are two methods for assessing groundwater vulnerability: intrinsic and specific.^[^ ^17^ ^]^ These methods are defined as follows by Zwahlen;^[^ ^18^ ^]^

Intrinsic vulnerability focuses on the travel time of a hypothetical fixed pollutant particle, which is affected by geological, and hydrogeological conditions, regardless of its type and pollution scenario.^[^ ^18^ ^]^ On the other hand, specific vulnerability completes the intrinsic vulnerability assessment by considering the characteristics of a pollutant and the changes it can undergo.^[^ ^18^ ^]^ It depends on the interaction of a pollutant or group of pollutants with various components of the natural system.^[^ ^19^ ^]^

Literature indicates that intrinsic groundwater vulnerability analysis is the most frequently employed method in vulnerability mapping studies, as it does not necessitate data on the degradation processes of pollutants between layers.^[^ ^20^ ^]^

Groundwater vulnerability can be categorized into three main approaches: index overlay methods, statistical techniques, and process‐based algorithms.^[^ ^16^, ^21^, ^22^ ^]^ Statistical Techniques: These involve methods like Logistic Regression Model, Discriminant Analysis, Cluster Analysis, Principal Component Analysis, Contaminant Modeling, and Geostatistical Assessments. These methods assess groundwater vulnerability by analyzing data. Contaminant modeling or geostatistical assessments are employed to predict the risk of groundwater contamination. These methods determine vulnerability by creating data‐based models. Process‐Based Methods: These encompass approaches like the Behavior Assessment Model, Attenuation Factor, and Meta Model. These methods rely on understanding groundwater movement and interactions. Approaches such as the Behavior Assessment Model and the Attenuation Factor fall into this category. The Meta Model provides a more comprehensive assessment by combining different methods. Index Overlay Methods: These include models such as DRASTIC, GOD, SINTACS, EPIC, PI, COP, IRISH, AVI, and SI. These methods combine specific parameters using an index or scoring system. These scores are used to assess the potential for groundwater contamination. DRASTIC is the most preferred and used index method. Each parameter is multiplied by a specific weight, and the results are summed. DRASTIC is useful for mapping the potential for groundwater contamination. The index overlay methods are popular due to their simplicity and minimal data requirements.^[^ ^14^, ^23^, ^24^ ^]^

The aforementioned methods are used to assess groundwater vulnerability in aquifer systems at the basin, catchment, or watershed scale, especially when sufficient data is lacking. The selection of models incorporating these methods varies based on ease of use, cost of obtaining the models, and computational simplicity. Among these methods, DRASTIC is the most widely used due to its ease of application, the widespread availability of required data, and its accessibility. The DRASTIC method is suitable for application in all regions of the world.^[^ ^15^, ^25^ ^]^

In the DRASTIC method, the interrelationships between various parameters ensure that none are omitted, resulting in accurate statistical outcomes.^[^ ^26^ ^]^ The DRASTIC model derives its name from the seven key parameters used to determine groundwater vulnerability. These parameters: Depth to Water (D), Net Recharge (R), Aquifer Media (A), Soil Media (S), Topography (T), Impact of the Vadose Zone (I), and Hydraulic Conductivity of the Aquifer (C). Each of these hydrogeological features or parameters is weighted based on its impact on groundwater vulnerability and classified into ranges, with each range assigned a numerical rating. This allows for the determination and analysis of the groundwater vulnerability index within a GIS environment.^[^ ^5^, ^16^, ^27^ ^]^ Developing GIS‐based vulnerability maps is important for effective aquifer management. The algebraic operations used in mapping allow for mathematical operations between each layer, resulting in spatial vulnerability maps. GIS provides a reliable environment for the management, analysis, and visualization of spatial, temporal, and nonspatial data sets.

Therefore, GIS‐based maps are an important tool for processing multilayered modeling environments and data sets. The DRASTIC model is frequently used in groundwater vulnerability analysis with GIS in basins.^[^ ^28^, ^29^, ^30^, ^31^ ^]^ Many researchers have effectively mapped groundwater vulnerability zones using the layers of the seven parameters that form the basis of the DRASTIC model through GIS.^[^ ^14^, ^15^, ^23^, ^24^, ^32^, ^33^, ^34^, ^35^, ^36^, ^37^, ^38^, ^39^, ^40^, ^41^ ^]^ The vulnerability maps typically categorize regions into vulnerability levels ranging from very low to very high.^[^ ^28^, ^29^, ^31^ ^]^ The DRASTIC model is often validated using water quality indicators such as nitrate concentrations.^[^ ^29^, ^31^ ^]^ These vulnerability maps are valuable tools for groundwater management, pollution control, and land‐use planning in different areas.^[^ ^28^, ^29^, ^30^, ^42^, ^43^ ^]^

This study created a vulnerability map of groundwater resources in the Kesikköprü Baraj Lake Basin using the GIS‐based DRASTIC index model. This lake supplies drinking water to Ankara province during drought periods.

Located in the southeastern region of Ankara, Kesikköprü Dam is one of the 11 dams built along the Kızılırmak River, ≈120 km from the city of Ankara. The dam was constructed between 1959 and 1966. Water extracted from Kesikköprü Dam is conveyed to the İvedik Drinking Water Treatment Facility through three separate pipelines totaling 128 km in length. These pipelines were installed between 2007 and 2008.^[^ ^44^ ^]^ Despite the availability of sufficient water in Ankara's dams to meet the city's needs, the initial segment of the Kızılırmak water supply system was not utilized from February 2009 to 2014. Starting in June 2014, water from Kesikköprü Dam has been mixed with water from other dams before being sent for treatment at the İvedik Drinking Water Treatment Plant. Following treatment, this water is distributed through the network to meet the water requirements of Ankara province and its districts, as reported by Ankara Metropolitan Water and Sewerage Works General Directorate.^[^ ^45^ ^]^ Kesikköprü Dam, in combination with the first segment of the Kızılırmak water supply system, acts as a safeguard for Ankara against drought. With a total of 384 km of pipelines in place, the dam is capable of meeting Ankara's water needs for the next two decades.^[^ ^44^, ^46^ ^]^

Cebe and Gülçiçek Uysal (2024) identified diffuse pollution sources in the Kesikköprü Dam Lake Basin, including agricultural activities, urbanization, livestock, leaking septic tanks, and atmospheric deposition. They calculated total nitrogen (TN) and total phosphorus (TP) loads, noting significant pollution increases in areas with intensive anthropogenic activities.^[^ ^46^ ^]^ In another study, Gülçiçek Uysal & Cebe (2024) projected the rise in point source pollution due to population growth between 2022 and 2050, emphasizing the anticipated deterioration of water quality without intervention.^[^ ^47^ ^]^

Complementary research by Gülçiçek Uysal and Cebe (2023) employed a multicriteria evaluation method (Analytical Hierarchy Process) to develop a sensitivity map, revealing that settlements, septic systems, and mining sites are located in zones categorized as “Very High Sensitivity” and “Medium Sensitivity.”^[^ ^48^ ^]^

Additionally, Yurttaş (2023) conducted a hydrogeochemical and isotopic assessment of surface and groundwater sources around the Kesikköprü Dam. His analyses indicated that pollution primarily stems from agricultural and geogenic origins.^[^ ^49^ ^]^

The Water Framework Directive, adopted by the European Union in 2000, establishes a framework for member countries to protect and enhance water quality while ensuring the sustainable management of water resources. The European Water Framework Directive has effectively promoted an integrated approach to water management at the basin level, and the Republic of Turkey has embraced this directive as a guiding framework. In 2010, Turkey implemented various regulations based on the Water Framework Directive to enhance the management of its water resources. In this context, integrated water resources management policies have been developed through documents such as the Turkey Water Management Strategy Document and Action Plan. By adopting integrated water resources management, Turkey is working to implement the principles of the Water Framework Directive and aims for a sustainable future by utilizing water resources more efficiently.^[^ ^50^, ^51^ ^]^ Integrated water resources management (IWRM), is indeed crucial for effective water resource management worldwide. The primary goal of IWRM is to harmonize water resources used for various purposes (such as drinking water supply and energy production) to ensure their smooth coexistence.^[^ ^52^ ^]^

In this study, the groundwater vulnerability of the Kesikköprü Dam Lake Basin—a critical drinking water resource for Ankara, the capital city of Türkiye—was evaluated using the DRASTIC model. Model outputs were statistically validated using nitrate and total organic carbon (TOC) concentrations, which are widely used indicators in the groundwater vulnerability literature. While the methodology itself follows established approaches, the key contribution of this study lies in the spatial integration of anthropogenic pressures with vulnerability zones, enabling a more comprehensive interpretation of both natural and human‐induced factors. The findings provide valuable insights for decision‐making in groundwater protection by allowing the evaluation of stressors, such as urban settlements, agricultural activities, and mining operations in relation to the vulnerability classifications. This approach offers practical contributions to the literature on sustainable groundwater management in semiarid basins, particularly under the mounting challenges posed by climate change, water scarcity, and pollution pressures.

## 2 Experimental Section

### 2.1 Study Area

**Figure 1:**
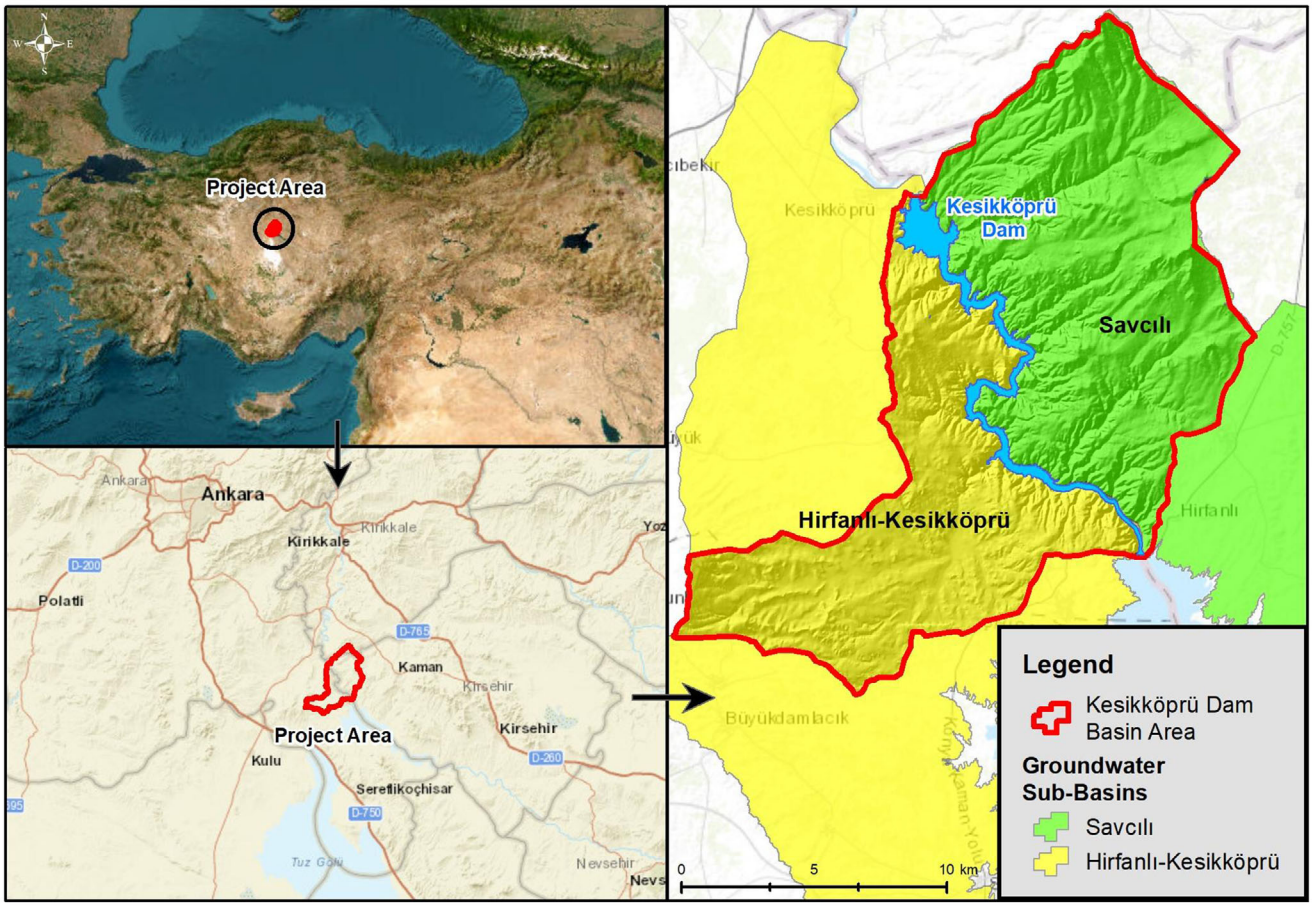
Location map of the Kesikköprü Dam Lake Basin.^[^ ^53^ ^]^

Spanning a length of 1151 km, the Kızılırmak River is Türkiye's longest river and covers a vast drainage area encompassing 82 181 km^2^. Situated in the eastern part of the Central Anatolia Region, the Kızılırmak Basin stands as the second‐largest basin within Türkiye.^[^ ^48^ ^]^ The Kesikköprü Dam Lake Basin is located within 33°23′ to 33°30′ east longitudes and 39°13′ to 39°27′ north latitudes. The entire study area encompassing the Kesikköprü Dam Lake Basin covers a total area of 252 km^2^. 104.92 km^2^ of the study area lies within the provincial boundaries of Ankara, specifically in the Bala and Şereflikoçhisar districts. 138.84 km^2^ of the study area lies within the Kaman district, situated within the provincial borders of Kırşehir, while the remaining 8.66 km^2^ are located within the jurisdiction of the Çelebi district in the provincial borders of Kırıkkale (**Figure** **1**).

The region to the east of the Kızılırmak River, which divides the Kesikköprü Dam Basin in two, features a climate marked by hot summers and cold, snowy winters. Due to its elevation, it ranks among the coldest areas of Ankara during the winter months. Although summers are hot and mild, it is noted that even in the driest months, precipitation levels are significantly higher than in other regions. In the areas west of the river, summers are mild, dry, and clear, while winters are extremely cold and snowy.^[^ ^48^ ^]^

#### 2.1.1 Anthropogenic Activities in the Basin

**Figure 2:**
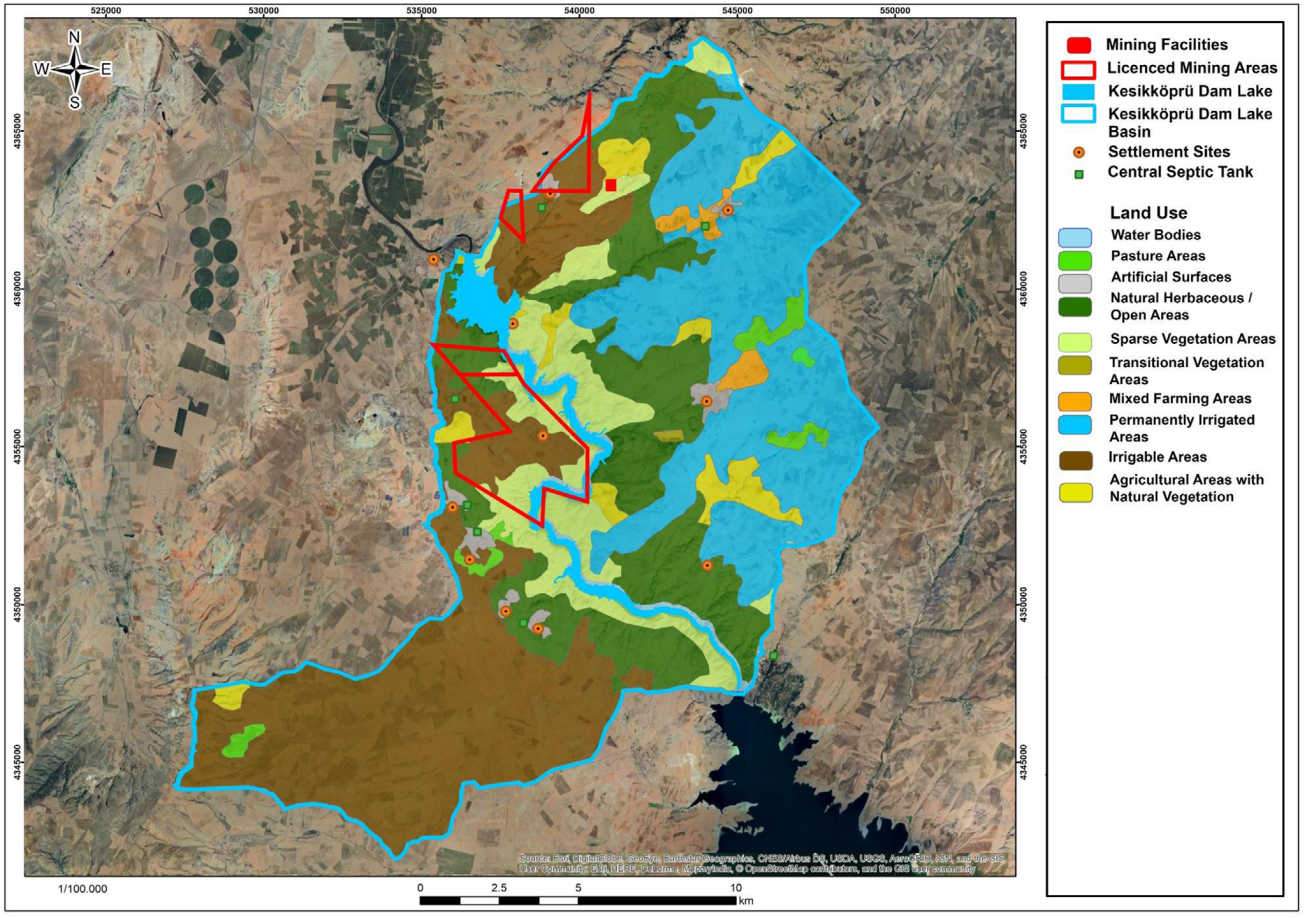
Map showing the settlements, the active mining site, and the land use within the Kesikköprü Dam Lake Basin.

The primary sources of pollution that could adversely affect water resources in the Kesikköprü Dam Lake Basin are domestic wastewater and agricultural activities. A total of 13 rural settlements are located within the Kesikköprü Dam Lake Basin (**Figure** **2**). In the Bala district, which includes the villages of Kesikköprü, Tepeköy, Küçükbıyık, Küçükcamili, Büyükcamili, and Bektaşlı, drinking and domestic water needs are met by the Bala Tepeköy Water Treatment Plant, operated by the Ankara Water and Sewerage Administration (ASKİ). In contrast, water supply for the seven settlements located within the Kaman district—namely Ağapınar, Karaosman, Buğuz, Tatık, Kargınyenice, İkizler, and Hirfanlı—is provided through groundwater resources. Approximately 40% of the total population in the study area (1573 out of 3822 people) relies on groundwater for drinking and domestic purposes.^[^ ^48^ ^]^

When the sewerage and septic tank status of the settlements in the study area was examined, it was determined that the wastewater of 11 settlements was transmitted to central septic tanks via the sewerage network, the wastewater of 1 settlement (Kargınyenice village with a population of 816 people) was discharged directly into the Kızılırmak River, and the wastewater of 1 settlement (İkizler village with a population of 74 people) was collected in septic tanks. The wastewater collected in the central septic tanks is removed from the basin area by the municipality with the help of a sewage tanker at certain intervals.

Agricultural activities conducted in the Kesikköprü Dam Lake Basin represent one of the most significant sources of income in the area. To assess the land use status in the Kesikköprü Dam Basin, the Coordination of Information on the Environment CORINE data from the Ministry of Agriculture and Forestry of the Republic of Turkey was utilized. CORINE is the land cover/use data generated through computer‐aided visual interpretation of satellite images, following the Land Cover/Use Classification established by the European Environment Agency (EEA). The EEA oversees the same fundamental data and develops a standard database across all EEA member countries, adhering to the criteria and classification system it defines, to identify environmental changes in the land, manage natural resources effectively, and formulate environmental policies.^[^ ^54^ ^]^ A land use map of the Kesikköprü Dam Basin was created using the 2018 CORINE data from the Ministry of Agriculture and Forestry and is shown in Figure 2. When the land use map of the basin (Figure 2) is examined, it is clearly seen that the region predominantly consists of natural herbaceous and agricultural lands. The distribution of land use within the dam recharge basin is as follows: water bodies cover 460 hectares, pasture areas 435 hectares, artificial areas 330 hectares, natural meadows 5180 hectares, sparsely vegetated areas 2760 hectares, transitional vegetation areas 28 hectares, mixed agricultural areas 265 hectares, permanently irrigated areas 6370 hectares, and agricultural areas with natural vegetation 1035 hectares.

There are no large‐scale livestock enterprises in the Kesikköprü Dam Lake Basin. Generally, livestock activities in the region consist of animals raised by individuals in their own barns and coops.

There is one open‐pit iron mining operation in the Kesikköprü Dam Lake Basin. The active iron mine is located on granite rock classified as low permeable/impermeable. There is no waste storage facility or enrichment facility for processing mining waste. According to the operation's statements, there are no activities that would cause pollution of water resources through surface runoff, and the impermeable bases of the waste areas prevent any future pollution from these regions (Figure 2).

### 2.2 Hydrogeology

The study area is geologically situated within the Central Anatolian Crystalline Complex, particularly along the boundary between the Kırşehir Massif and the Central Anatolian Fault Zone. The region exhibits a diverse assemblage of lithologies including Paleozoic‐aged metamorphic rocks and marbles, Mesozoic‐aged ophiolitic mélanges and granitoids, and Neogene—Quaternary sedimentary formations. This complex tectonic and lithological setting plays a significant role in shaping the permeability characteristics and groundwater flow paths across the Kesikköprü Dam Basin.^[^ ^44^, ^49^ ^]^

Previous geological and hydrogeological investigations in the area were conducted by the General Directorate of State Hydraulic Works (DSI)^[^ ^44^, ^55^ ^]^ and by Yurttaş (2023)^[^ ^49^ ^]^ who provided a comprehensive assessment of the stratigraphy, hydrostratigraphic units, aquifer characteristics, and groundwater quality across the region. These studies formed the basis for delineating sub‐basin boundaries, recharge zones, and aquifer classifications in this study. Kesikköprü Dam Lake receives its water supply from several sources, including the Kızılırmak River, Büyük Öz, Çenger, and Aydın streams. Within the study area, there are two primary groundwater sub‐basins: the Hirfanlı–Kesikköprü groundwater sub‐basin and the Savaşlı groundwater sub‐basin (Figure 1).

The Hirfanlı–Kesikköprü groundwater sub‐basin, situated in the western part of the Kesikköprü Dam Basin, encompasses the catchment area on the Ankara side of the Hirfanlı and Kesikköprü Dam lakes (Figure 1). Within the Hirfanlı–Kesikköprü groundwater sub‐basin, there are various geological units, including Paleozoic‐aged marbles (P2) and metamorphic series (P1), Mesozoic‐aged granite‐granodiorite (Gr) and ophiolitic mélange (Mof), Neogene‐aged unseparated terrestrial sediments (n2) and limestone (n3), Eocene flysch (ef), and quaternary alluvium (Qal) formations, which are observed throughout the region.^[^ ^44^ ^]^

**Figure 3:**
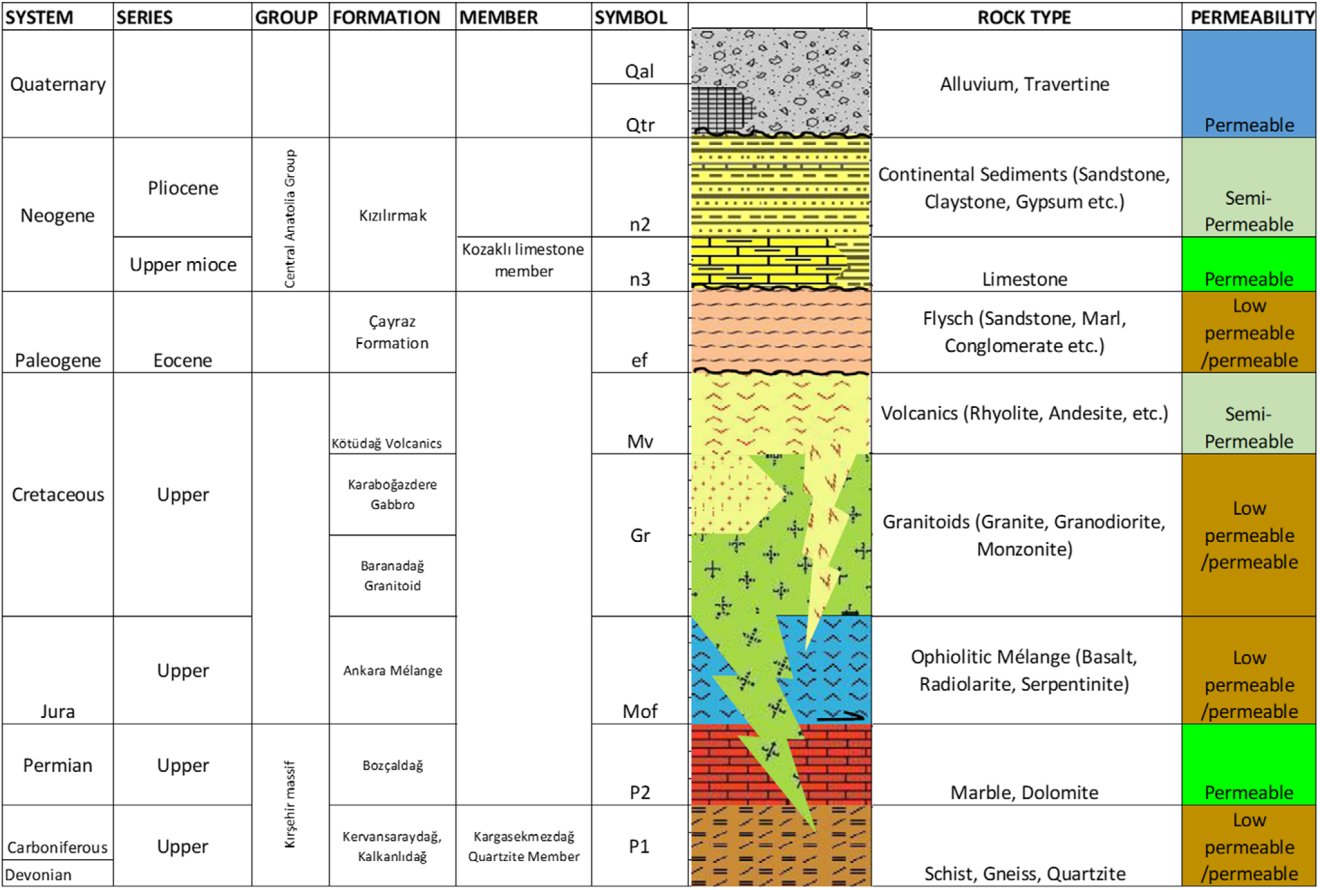
Unscaled stratigraphic column showing the vertical distribution of geological formations and permeability.^[^ ^49^ ^]^

The Savaşlı groundwater sub‐basin, which encompasses the Kırşehir province in the eastern section of the Kesikköprü Dam Basin, comprises various geological formations, including Paleozoic‐aged marbles (P2) and metamorphic series (P1), Mesozoic‐aged granite‐granodiorite (Gr), Volcanics (Mv), and ophiolitic mélange (Mof), as well as Neogene‐aged unseparated terrestrial sediments (n2), Quaternary travertine (Qtr), and Quaternary alluvium (Qal) units region.^[^ ^44^ ^]^ The regional distribution of the geological units and their permeability characteristics are shown in **Figure** **3**.

**Figure 4:**
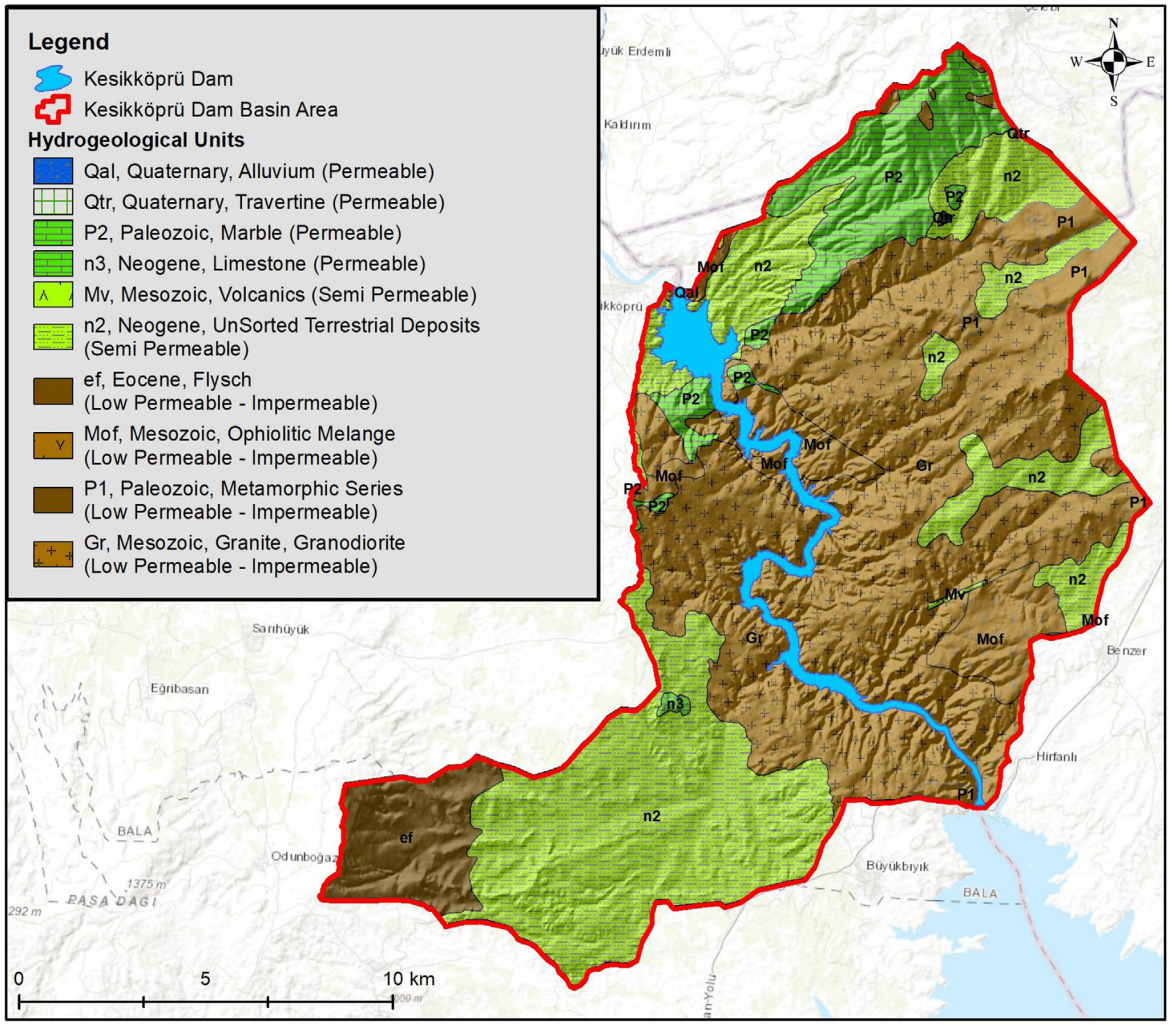
Map displaying the permeability characteristics of geological units in the Kesikköprü Dam Lake Basin.

Permeability properties of the units are specified in Figure 3. Blue‐colored units represent primary permeable units, green‐colored units, permeable and semipermeable units with secondary porosity, and brown units represent less permeable units. The primary porosity is the porosity of the rock since the formation of rock. Secondary porosity is the porosity structure caused by chemical processes such as physical and rock dissolves such as fractures, cracks over time.^[^ ^56^ ^]^ Hydrogeology map of the study area is presented in **Figure** **4**.

As illustrated in Figures 3 and 4, the Kesikköprü Dam Basin comprises a diverse range of geological units with distinct hydrogeological characteristics that influence groundwater flow and storage. The spatial distribution of formations, such as Quaternary alluvium (Qal), travertine (Qtr), and limestones (n3) are predominantly located in valley bottoms and near stream networks, and are categorized as highly permeable, playing a crucial role in groundwater recharge and transmission. These zones function as the main unconfined aquifers of the region. In contrast, formations such as the Eocene flysch (ef), ophiolitic mélange (Mof), and granite‐granodiorite (Gr) exhibit low to very low permeability, often acting as aquitards or aquicludes, limiting vertical and lateral groundwater movement. These units are generally located in the southern and central sections of the basin. The Paleozoic metamorphic series (P1) and marbles (P2), while generally of low permeability, may support localized flow through fractured or karstified zones, depending on the structural setting. The Neogene sediments (n2) and volcanic rocks (Mv) are characterized by semipermeable behavior, contributing to intermediate recharge zones where their heterogeneity allows limited groundwater infiltration and storage. These formations dominate the central and northern parts of the basin and represent transitional zones between high‐permeability alluvium and low‐permeability basement rocks.^[^ ^49^ ^]^

### 2.3 Defining Parameters for the DRASTIC Model

The DRASTIC model methodology has been widely employed by researchers globally for assessing groundwater vulnerability. The DRASTIC model, first developed by Aller et al., (1987)^[^ ^27^ ^]^ under the sponsorship of the United States Environmental Protection Agency (US EPA), represents a standardized and widely adopted system for evaluating groundwater pollution potential based on hydrogeologic settings. Fundamentally, this model relies on seven key parameters to estimate vulnerability. These parameters collectively contribute to the computation of the DRASTIC index value, as outlined by.^[^ ^57^ ^]^ The DRASTIC parameters are depth of water (D), net recharge (R), aquifer properties (A), soil characteristics (S), topographical features (T), vadose zone effects (I), and conductivity (C), as detailed by.^[^ ^58^ ^]^ It is important to note that both the proportions and weights assigned to these seven parameters are adapted to reflect the specific local conditions. Equation (1) is utilized to derive the DRASTIC index value

(1) $$\begin{array}{ccc} DRASTICIndex\left(Di\right) & = & DrDw+RrRw+ArAw+SrSw\\ & & +TrTw+IrIw+CrCw \end{array}$$

The DRASTIC parameters are categorized based on different hydrological environments, and their respective scores reflect their capacity to facilitate pollutant transport within those environments. The assigned weights for these seven factors vary from 1 to 5, indicating the relative significance of each parameter. The DRASTIC index (Di) is used to assess groundwater vulnerability by partitioning the area. It is calculated as the sum of the product of predicted ratings (subscript “r”) and the corresponding weights (subscript “w”) assigned to each of the seven data layers. This method was described by Pacheco et al. (2015).^[^ ^59^ ^]^

The studies were carried out to assign accurate spatial scores to the seven DRASTIC parameters (D, R, A, S, T, I, C) by using GIS tools and combining the weighted scores to obtain the DRASTIC Index, which exposes the groundwater vulnerability to conduct a spatial risk analysis for pollution.

#### 2.3.1 Water Table Depth (D)

The depth to the water table is a fundamental parameter in assessing groundwater vulnerability, as it directly influences the time available for pollutant attenuation processes, such as dispersion, oxidation, and biodegradation. A shallower water table depth is typically associated with higher vulnerability scores within the DRASTIC framework, as contaminants have less opportunity to be naturally filtered or diluted before reaching the aquifer.

In the study area, the number of available observation wells was insufficient to capture the spatial variability of groundwater levels across the entire 25 242‐hectare basin. To address this limitation, and based on guidance from the State Hydraulic Works (DSİ), natural water discharge points such as springs and fountains were used as alternative reference indicators of groundwater level. This approach also allowed the incorporation of local topographic features and stream networks to infer the groundwater surface.

To construct a continuous groundwater surface, the Kriging interpolation method was employed. Kriging is a geostatistical technique that not only considers the distance between known data points but also their spatial autocorrelation, which provides more accurate estimations than deterministic methods such as Inverse Distance Weighting (IDW), particularly in heterogeneous terrains.^[^ ^60^ ^]^ Given that groundwater levels are spatially correlated and influenced by geological and topographic features, Kriging was deemed the most appropriate interpolation method for this study.

**Figure 5:**
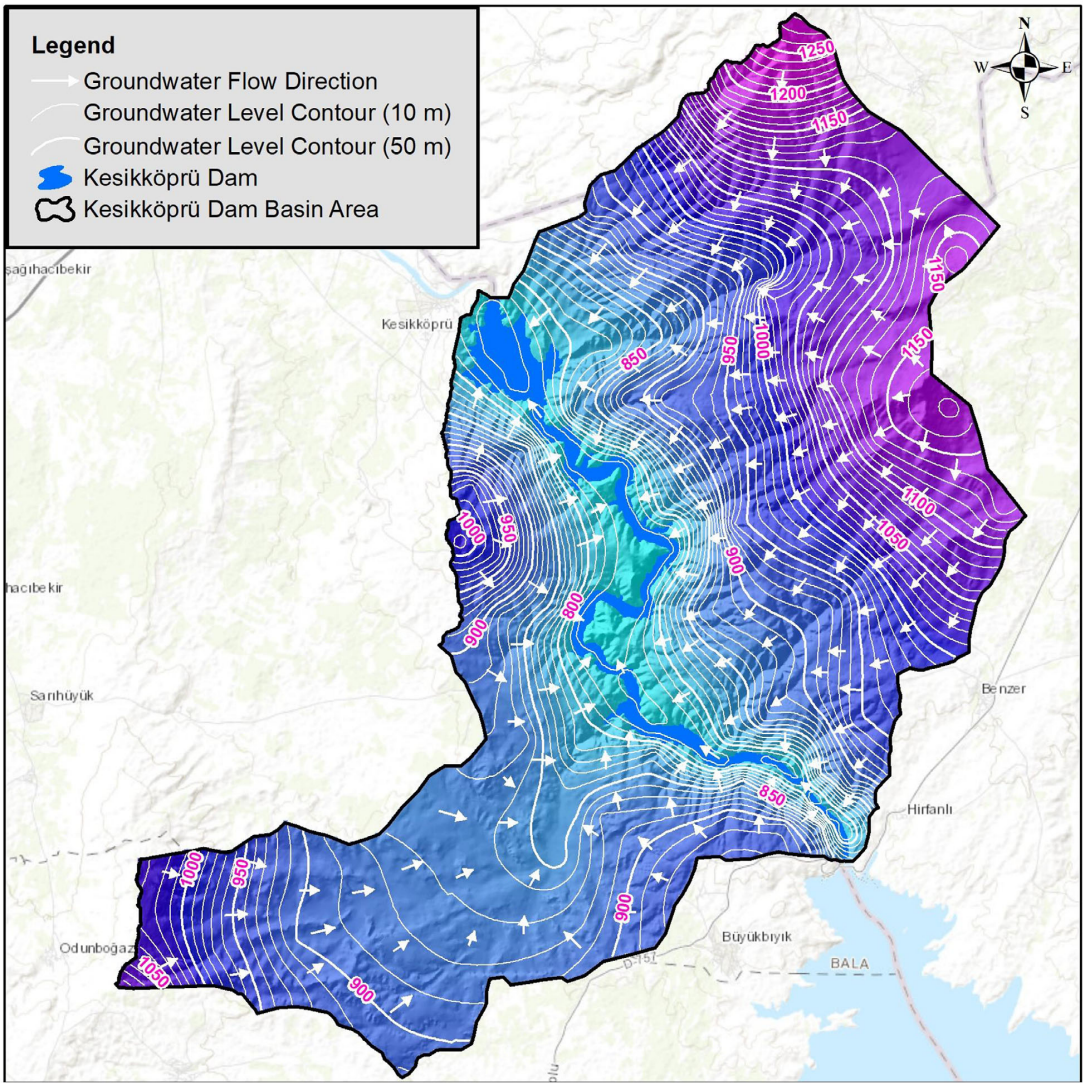
Map illustrating the groundwater table depths and flow directions.

The groundwater elevation contours were generated with 0‐m intervals using the interpolated surface, as illustrated in **Figure** **5**. These contours indicate that groundwater flows from high‐elevation zones to lower areas, consistent with the topographic slope. Specifically, the hydraulic gradient in the western part of the basin flows eastward, while in the eastern part it flows westward, ultimately converging toward the reservoir and the main river channel. Water table depth values (D) are scored in raster format using the ranges specified below, and the raster data file is converted to an output in the form of pixels (**Table** **1**).

**Table 1:** Water table depth scoring criteria for DRASTIC model (D).

Water table depth score (D) D Water Table Depth Points (m)	
Water table depth (m)	Score (D)
0–1.52	10
1.52–4.57	9
4.57–9.14	7
9.14–15.24	5
15.24–22.86	3
22.86–30.48	2
30.48>	1

#### 2.3.2 Net Recharge (R)

Net recharge refers to the quantity of water that enters the aquifer. This value can be computed using available data on an annual or monthly basis. While recharge serves to dilute pollutants entering the aquifer, it also serves as the primary conduit for the transport of contaminants. Consequently, the level of vulnerability is positively correlated with the volume of net recharge. Calculating net recharge involves applying a mass balance to water mass using climate data.

A specific methodology and formulation were employed to calculate the recharge in the basin. In this context, the spatial distribution of precipitation was assessed, and infiltration coefficients were established based on the characteristics of the lithological units. Furthermore, while delineating the boundaries of the aquifer recharge areas, the hydrological conditions created by the water structures located upstream, the negative boundary conditions imposed by low‐permeability and impermeable rocks, and the positive boundary conditions established by permeable rocks were particularly considered. The following formula (Equation (2)) was utilized for the recharge calculation from lithological units in the developed groundwater recharge table

(2) $$Q=\left(A*P*I\right)/1000$$

where, *Q* is the net recharge from precipitation (hm^3^ year^−1^), *A* is area (km^2^), *P* is precipitation (mm), *I* is percolation coefficient.

The basin spans an extensive area of 25.242 hectares and encompasses a diverse range of 10 distinct lithologies. For the recharge calculation, a dataset comprising 62 years of precipitation data from the Kaman Meteorology Station, which is considered the most representative of the region, is utilized. The annual average precipitation value recorded at the Kaman Meteorology Station within the study area is determined to be 39.3 mm.

The net recharge calculation for the study area is performed on a lithology‐specific basis, with separate percolation coefficients assigned to each of the different lithological units. Accordingly: Quaternary Alluvium (Qal) and Quaternary Travertine (Qtr) comprise relatively fine‐grained material, and to err on the side of caution, an infiltration rate of 15% was applied.

Eocene Flysch (ef), Mesozoic Granite, Granodiorite (Gr), Mesozoic Ophiolitic Melange (Mof), and Paleozoic Metamorphic Series (P1) are regarded as low‐permeability to impermeable, so an infiltration rate of 1% was utilized. An inward flow rate of 10% was applied for the Paleozoic Marble (P2) unit. Neogene Undifferentiated Sediments (n2) also consist of clayey‐silty fine grained material, thus an infiltration rate of 5% was implemented. An infiltration rate of 5% was assigned to the Mesozoic Volcanics (Mv) units, while an infiltration rate of 10% was selected for the Neogene Limestone (n3) unit, which is classified as permeable consolidated.

The groundwater recharge table calculated by infiltration from lithological units for the Kesikköprü Dam recharge basin is provided below (**Table** **2**).^[^ ^49^ ^]^

**Table 2:** Calculated groundwater recharge values based on lithological units.

Lithology	Average precipitation (P) [mm]	Area (A) [km^2^]	Lithological percolation (I)	Net recharge from precipitation (Q) [hm^3^ year^−1^]
Qal	471.6	0.014	0.15	0.001
Qtr	471.6	0.13	0.15	0.009
n3	471.6	0.39	0.10	0.018
n2	471.6	78.99	0.05	1.863
ef	471.6	12.99	0.01	0.061
Mv	471.6	0.21	0.05	0.005
Mof	471.6	14.20	0.01	0.067
Gr	471.6	110.09	0.01	0.519
P2	471.6	20.81	0.10	0.981
P1	471.6	5.92	0.01	0.028
			Total	3.55

Following the creation of a data file containing net recharge values in raster format, the dataset is divided into 5 intervals to obtain net recharge scores for the DRASTIC model. These intervals are employed to reclassify the data files in raster format and assign a corresponding score, as outlined in **Table** **3**.

**Table 3:** Net recharge scoring criteria for DRASTIC model (R).

Net recharge score [R]	
Net recharge [cm]	Score (R)
0–5.08	1
5.08–10.16	3
10.16–17.78	6
17.78–25.4	8
25.4 >	9

#### 2.3.3 Aquifer Media (A)

The score for aquifer media is established by considering the permeability of each layer within the media. Higher permeability permits greater water flow, and consequently, it allows for a higher influx of pollutants into the aquifer. Thus, higher permeability results in a heightened degree of vulnerability in the assessment. The geological data used for this classification are obtained from the Kızılırmak Basin Master Plan prepared for DSI (General Directorate of State Hydraulic.^[^ ^44^ ^]^ The characteristics of the aquifers within the Kesikköprü Dam Lake Basin are divided into specific classes according to the DRASTIC score as indicated in **Table** **4**.

**Table 4:** Aquifer media scoring criteria based on geological formations (A).

Aquifer media score (A)	
Geological formation	Score
Massive shale	2
Metamorphic/volcanic	3
Eroded metamorphic/volcanic	4
Alternation of thin sandstone, limestone, and shale layers	6
Massive sandstone	6
Massive limestone	6
Sand and gravel	8
Basalt	9
Karst limestone	10

The corresponding score of each geological unit is assigned to a lithology polygon. This created layer is used to create a data file in raster format in terms of lithology type and DRASTIC score.

#### 2.3.4 Soil Media (S)

The characteristics of soil media have a significant impact on the movement of pollutants and water from the surface to the aquifer. It can influence the types of chemical reactions that take place due to the interactions between water and soil. For instance, the structure of the soil surface can influence sorption processes. Moreover, various types of soil can create more favorable conditions for microorganisms capable of biodegrading pollutants.

Major Soil Group (MSG) data used in soil classification in Türkiye has been converted to the Hydrological Soil Group (HSG) type defined in the DRASTIC method. “Landscape Character Analysis and Evaluation Technical Guide Supplementary Document, ” collaboratively prepared by the Ministry of Internal, the Ministry of Environment and Urbanization, the Ministry of Agriculture and Forestry, Ankara University and TÜBİTAK is used as a reference document.^[^ ^61^ ^]^

This study utilized the Runoff Curve Number Method (runoff Curve Number/CN), originally formulated by the United States Soil Conservation Service (now known as the United States Department of Agriculture Natural Resources Conservation Service), which employs water and soil characteristics to assess soil permeability. The Curve Number (CN) is a parameter employed for computing surface runoff during precipitation events. The CN value is determined according to the type of soil, the type of land use or vegetation, soil treatment methods, and hydrological conditions. A greater CN value corresponds to an increased surface runoff. In accordance with the CN approach devised by the Natural Resources Conservation Unit (formerly SCS), the soil conditions within the reservoir's watershed are categorized into hydrological soil classes, as detailed in **Table** **5**.^[^ ^62^ ^]^

**Table 5:** Hydrological soil groups (HSGs).^[^ ^63^ ^]^

Hydrological soil groups	Description
(Class A) Soils with low runoff (high infiltration) potential	Hydrologically, when fully wetted, soils with high infiltration and permeability rates have low runoff potential. Sandy soils containing less clay and silt are generally included in this group.
(Class B) Soils with less than moderate surface runoff Potential	Hydrologically, soils with moderate infiltration rate and permeability when fully wet are in this class. Surface runoff potential is moderate in soils consisting of a mixture of fine and coarse grains.
(Class C) Soils with higher than moderate surface flow Potential	Soils with very high levels of clay that have less than moderate infiltration and permeability when fully wet have a relatively high runoff potential.
(Class D) Soils with high runoff potential	There is a high level of runoff potential in soils that have lower infiltration and very low permeability when fully wet. Soils that contain a significant amount of clay and have an impermeable layer near the surface are generally in this class.

Hydrological soil groups of the study area are obtained by interpreting the data in the soil map database. By converting Large Soil Group (LSG) data to Hydrological Soil Group (HSG) data, HSG data are aligned with the suggested unit scale used in the DRASTIC methodology. The scores of the units are given below (**Table** **6**).

**Table 6:** Soil media scores used in the DRASTIC model.

Soil media scores		
Soil media	Score	HSG
None or very thin	10	—
Gravel	10	—
Annual	9	A
Organic soil	8	—
Clay that can absorb water and/or contains crushed stone	7	B
Sandy fertile soil	6	—
Loam	5	—
Silty fertile soil	4	C
Clay fertile soil	3	—
Mud	2	D
Clay (That does not absorb water and does not contain crushed stone)	1	—

#### 2.3.5 Topography (T)

The topographical features of the terrain play a significant role in groundwater vulnerability, primarily because the slope of the land is a critical factor in determining whether released contaminants will either flow or seep into the aquifer. On lower slopes, pollutants are less likely to be carried away by runoff and are, therefore, more inclined to percolate and infiltrate into the aquifer.

A digital elevation model (DEM) with a 10‐m resolution is employed in the study area to derive the surface slopes by using ArcGIS 10.8 spatial analysis tools. The surface slopes in the study area are expressed in percentage units. The scoring of the slope data aligns with the ranges provided in the DRASTIC methodology. These scores are then transformed into a raster data file utilizing the scoring scheme detailed in **Table** **7**.

**Table 7:** Topography scoring criteria based on slope percentages (T).

% Slope score (T)	
Interval [%]	Score (T)
0–2	10
2–6	9
6–12	5
12–18	3
18>	1

#### 2.3.6 Zone Effect (I)

The vadose zone represents the soil layer that is either unsaturated or intermittently saturated, positioned both above and below the water table. When the vadose zone exhibits high permeability, it will lead to a high degree of vulnerability for groundwater. The DRASTIC methodology involves categorizing geological units to assess their impact on the vadose zone concerning aquifers. Each lithology polygon is assigned a score based on the DRASTIC scoring criteria outlined in **Table** **8**. These scores are then used to create a raster dataset, effectively representing the impact of these geological features on the vadose zone and aquifers.

**Table 8:** Vadose zone effect scoring criteria based on geological formations (I).

Vadose zone effect score (I)	
Geological formation	Score
Silt/clay	1
Shale	3
Limestone	6
Sandstone	6
Stratified limestone, sandstone, shale	6
Sand and gravel containing significant amounts of clay and silt	6
Metamorphic/volcanic	4
Sand and gravel	8
Basalt	9
Karst limestone	10

#### 2.3.7 Hydraulic Conductivity (C)

Hydraulic conductivity is associated with the structures, bedding planes, and intergranular spaces within the aquifer. These elements serve as conduits for fluid movement and, correspondingly, for contaminant migration once pollutants enter the aquifer. A higher hydraulic conductivity is directly linked to an increased level of sensitivity and vulnerability to pollutants. Hydraulic conductivity coefficients for various geological units within the study area are estimated by conducting a literature review, as documented in Freeze and Cherry's study.^[^ ^56^ ^]^ Using these identified hydraulic conductivity values, each lithology polygon is assigned a score according to the DRASTIC classification categories provided in **Table** **9**. Subsequently, the vector data are transformed into a raster data file based on these scores.

**Table 9:** Hydraulic conductivity scoring criteria for DRASTIC model (C).

Hydraulic conductivity score (C)	
Unit [m day^−1^]	Score
0.04–4.07	1
4.07–12.21	2
12.21–28.49	4
28.49–40.7	6
40.7–81.4	8
81.4>	10

## 3 Results

### 3.1 DRASTIC Index for Groundwater Vulnerability

To obtain DRASTIC index for groundwater vulnerability, the raster data files that are prepared for the various DRASTIC input parameters are integrated using ArcGIS 10.8 software. The DRASTIC methodology implies assigning distinct weights to each input parameter, reflecting their significance in terms of groundwater vulnerability.^[^ ^64^ ^]^The weights for each parameter for the DRASTIC Index are in **Table** **10**.

**Table 10:** Weight values of DRASTIC parameters.^[^ ^27^ ^]^

DRASTIC parameter weights	
DRASTIC parameter	Weight
Water table depth (D)	5
Net recharge (R)	4
Aquifer media (A)	3
Soil media (S)	2
Topography (T)	1
Vadose zone effect (I)	5
Hydraulic conductivity (C)	3

**Figure 6:**
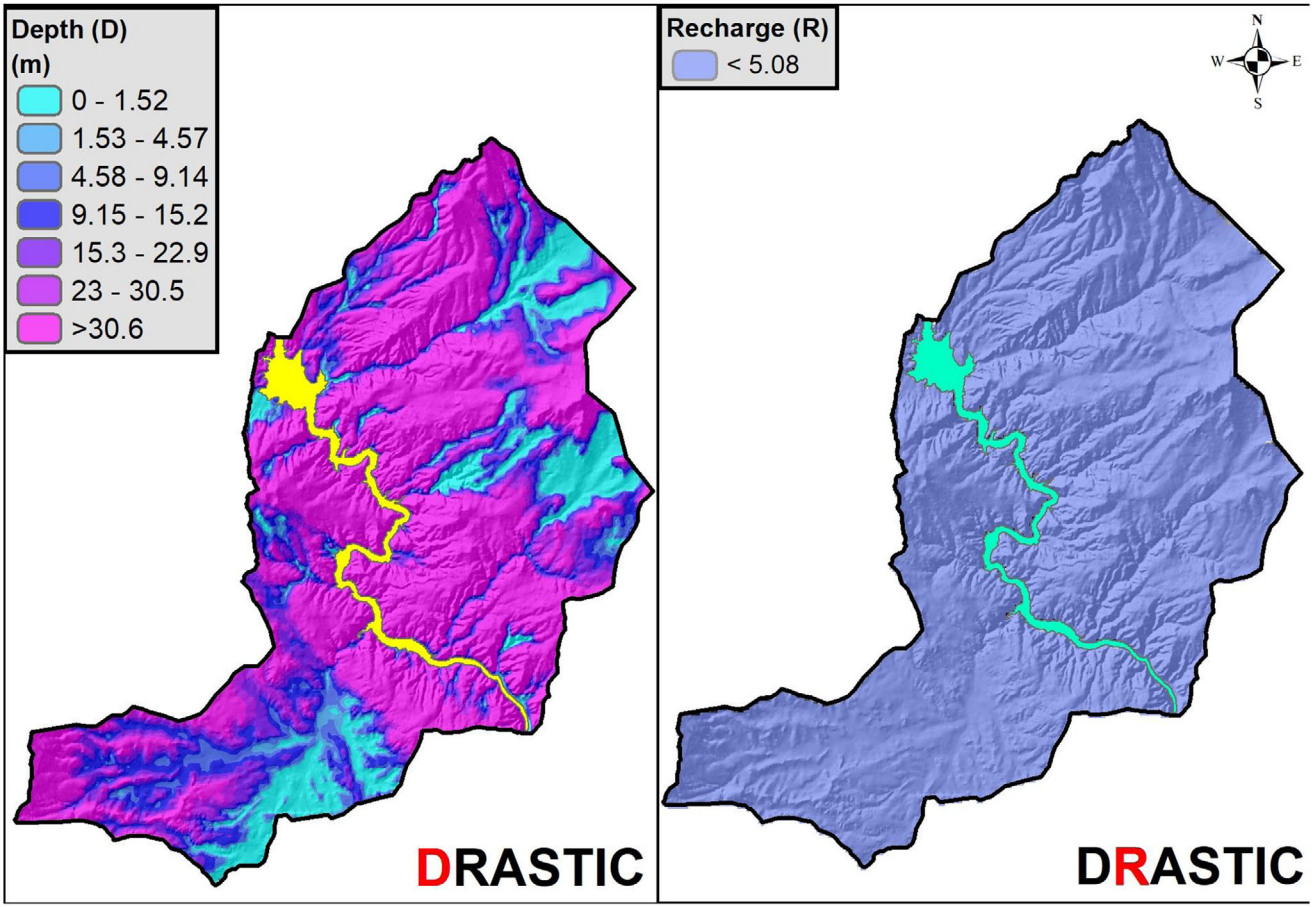
Spatial distribution maps of Depth to Water Table and Net Recharge parameters.

**Figure 7:**
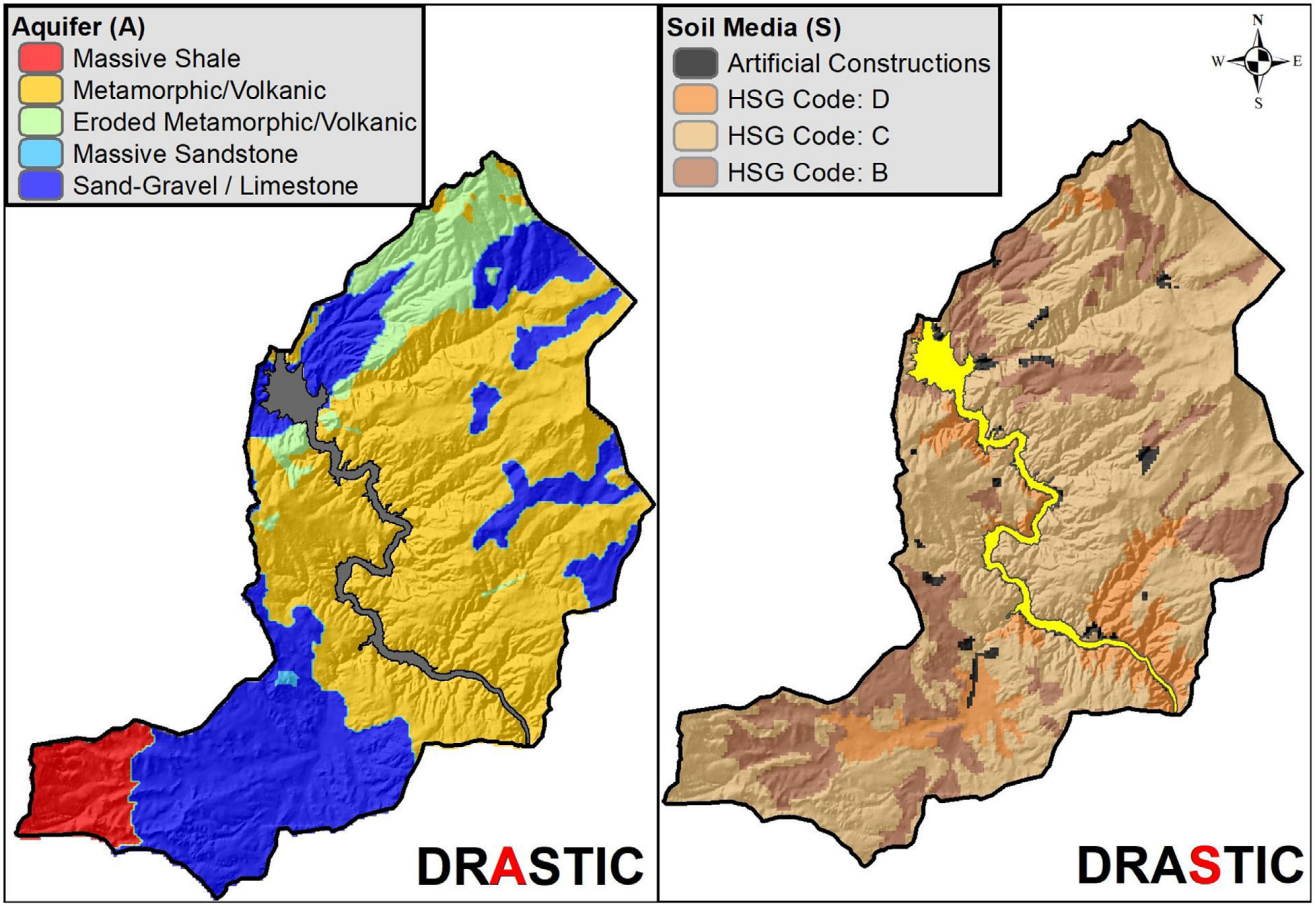
Spatial distribution maps of Aquifer Media and Soil Media parameters.

**Figure 8:**
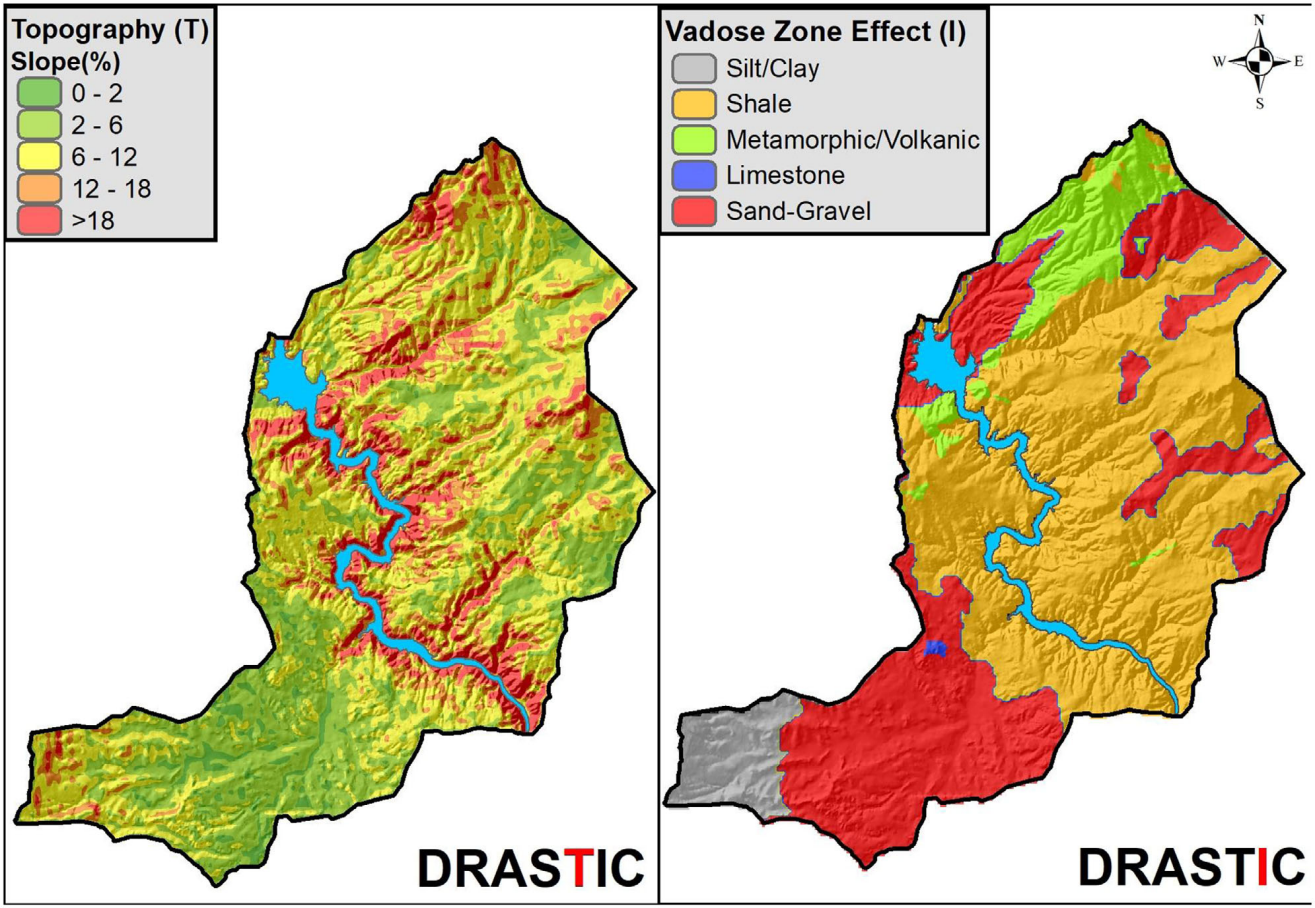
Spatial distribution maps of Topography and Vadose Zone parameters.

**Figure 9:**
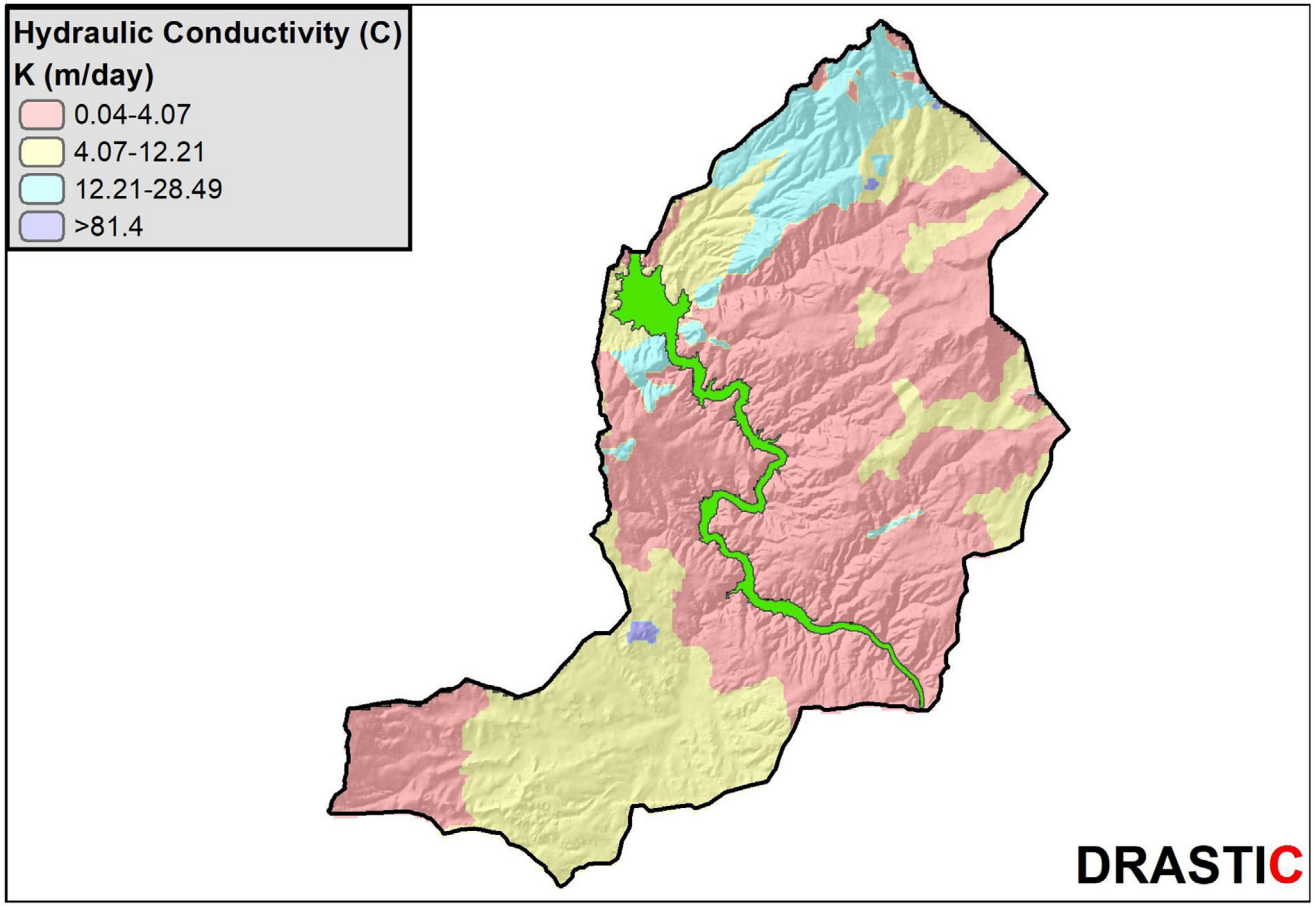
Spatial distribution map of Hydraulic Conductivity parameter.

The DRASTIC layer maps showing the spatial distributions of the scores for each of the 7 DRASTIC parameters (D, R, A, S, T, I, C) are created by using ArcGIS 10.8 (**Figures** **6**, **7**, **8**, **9**).

The scores for each of the seven DRASTIC parameters are merged with their respective weight coefficients using the GIS raster data calculation tool. Subsequently, to calculate the DRASTIC Index, the sum of the weighted scores for each point within the raster data file is created following the formula described in Equation (3)

(3) $$\begin{array}{ccc} DRASTICIndex\left(Di\right) & = & \left(Dx5\right)+\left(Rx4\right)+\left(Ax3\right)+\left(Sx2\right)\\ & & +\left(Tx1\right)+\left(Ix5\right)+\left(Cx3\right) \end{array}$$

The computing cell size used within the scope of the study is 100 × 100 m. The values obtained as output in the grid cells are categorized using the DRASTIC method vulnerability classification index, and each cell is assigned a color to that risk class as indicated below (**Table** **11**).

**Table 11:** DRASTIC vulnerability class color codes.^[^ ^27^ ^]^

Color codes		DRASTIC vulnerability class
<79	Pink	Very low
80–99	Light blue	Low
100–119	Blue	
120–139	Dark green	Medium
140–159	Light green	
160–179	Yellow	High
180–199	Orange	
>200	Red	Very high

### 3.2 Groundwater Risk Assessment

**Figure 10:**
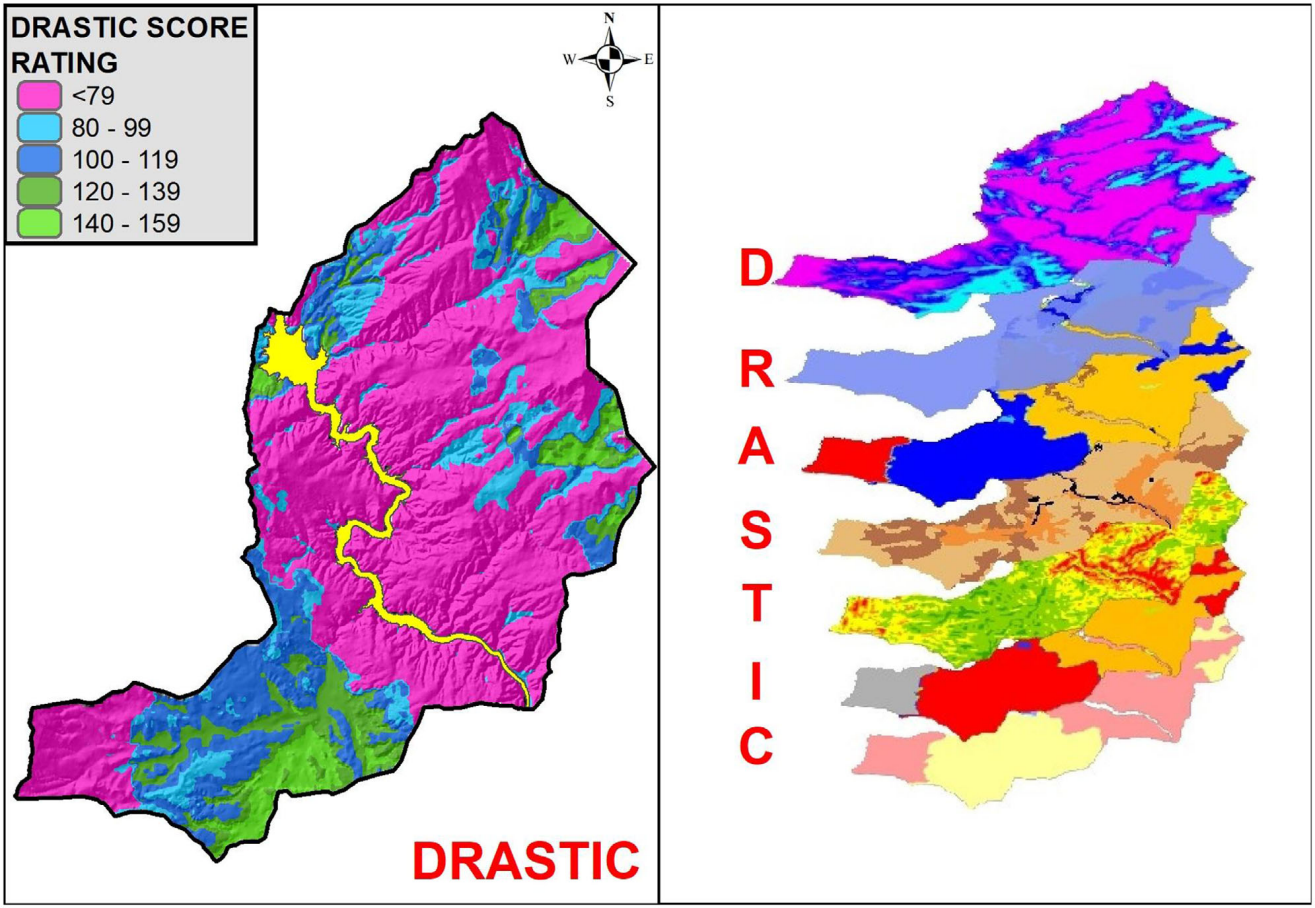
Groundwater vulnerability map classified by DRASTIC scores.

The risk analysis conducted within the dam lake basin area has categorized groundwater vulnerability into three class: “very low,” “low,” and “medium” (as shown in **Figure** **10**). A thorough explanation of the vulnerability classes, along with their corresponding definitions, is presented in **Table** **12**. The Kesikköprü Dam Lake basin does not exhibit significant “very high” or “high” groundwater risk. Areas near the southwestern and east‐northeastern borders of the basin fall into the “medium” vulnerability category. However, the majority of the study area is classified as having “very low” to “low” vulnerability. This determination of “very low” vulnerability is primarily due to the prevalence of low‐permeable and impermeable geological units, along with low net recharge rates resulting from geological and soil characteristics. Overall, the predominance of “low” vulnerability scores across the majority of the study area suggests a generally limited risk to groundwater quality under current conditions. This implies that the infiltration of contaminants would only pose a significant threat if pollutants—particularly conservative ones—are persistently and extensively introduced into the subsurface (Figure 10).

**Table 12:** Definition of a groundwater vulnerability class.

Vulnerability class	Definition
Medium	The presence of certain contaminants can increase groundwater vulnerability, provided that they are persistently introduced into the subsurface through regular leaching or surface discharge.
Low	Groundwater systems are at risk from conservative pollutants primarily when continuous and large‐scale emissions occur over an extended period.
Very low	Vertical groundwater movement through the confining units appears to be negligible, suggesting that these layers effectively restrict vertical leakage.

### 3.3 Assessment of Anthropogenic Activities Based on Groundwater Vulnerability Zones

**Figure 11:**
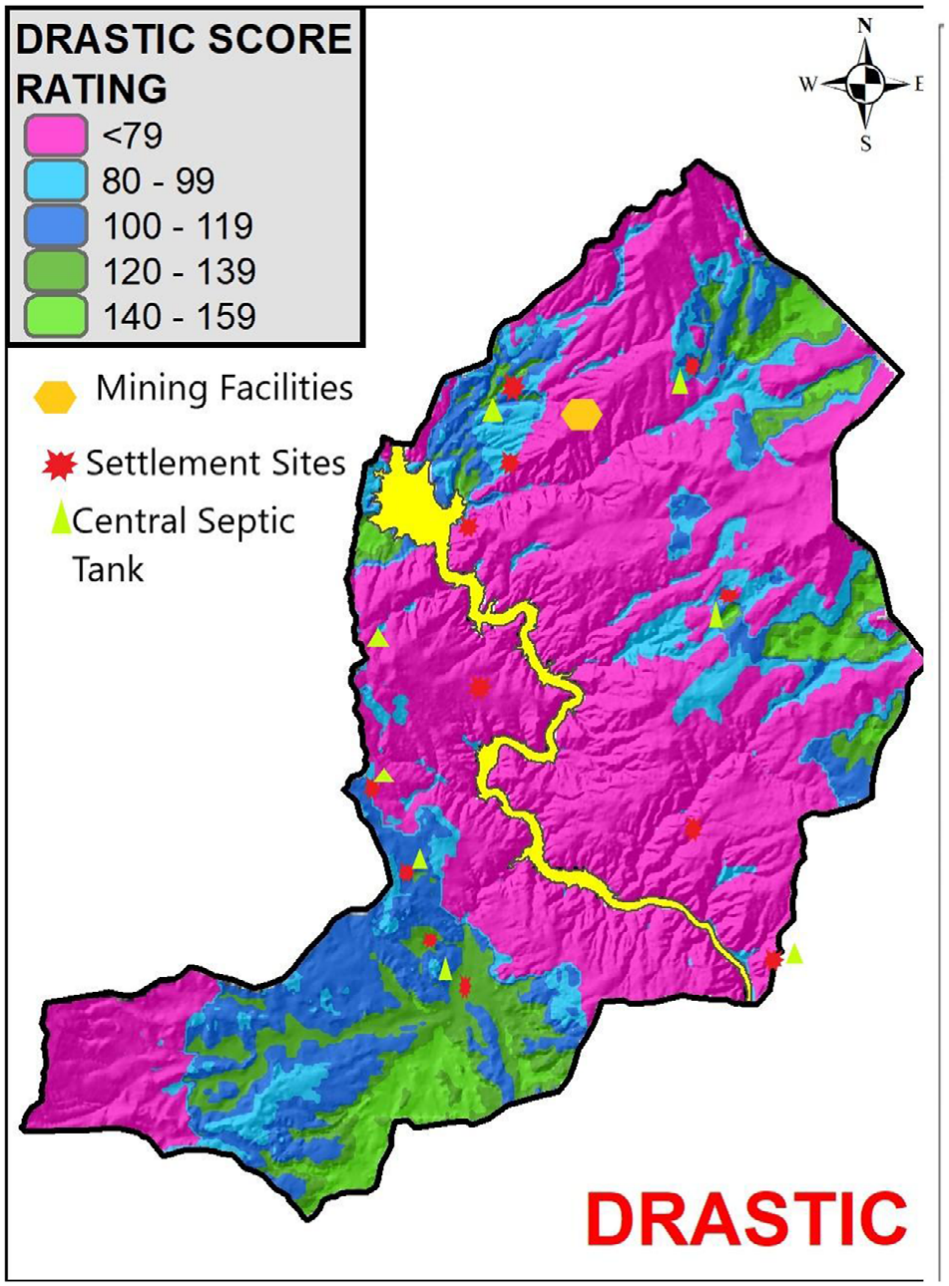
Spatial distribution of anthropogenic activities over groundwater vulnerability zones classified by the DRASTIC model.

Agricultural activities within the Kesikköprü Dam Lake Basin are predominantly located in areas classified as “very low” vulnerability zones on the DRASTIC map (**Figure** **11**). This spatial distribution suggests that, under current hydrogeological conditions, the potential for contaminants originating from agricultural practices—such as fertilizers, pesticides, and livestock waste—to infiltrate into the groundwater system remains relatively limited. The low permeability of the underlying lithological units and shallow groundwater recharge in these zones act as natural protective barriers. However, it is important to note that the cumulative and long‐term application of agrochemicals may still pose risks, particularly if land use patterns intensify or protective layers are disturbed.

The open‐pit iron mining site, is located entirely within a “very low” vulnerability zone as identified by the DRASTIC model (Figure 11). This spatial alignment validates the model output, given that the mine is situated on low‐permeability granite bedrock, and no waste storage or enrichment facilities exist on site. According to operational declarations, no direct discharge or runoff from mining activities occurs, and the impermeable base of the waste areas serves as an effective barrier. These findings suggest that, under current conditions, the mining activity does not pose a significant threat to groundwater resources, supporting the validity of the vulnerability classification.

As detailed in the Study Area section, the majority of rural settlements within the Kesikköprü Dam Lake Basin discharge domestic wastewater into centralized septic tanks, while two villages either release wastewater directly into surface water (Kargınyenice) or utilize individual septic systems (İkizler). Despite the formal infrastructure in place, field observations indicate poor maintenance of these systems, which may compromise their effectiveness. When these settlements are overlaid on the DRASTIC vulnerability map (Figure 11), it is evident that they are predominantly situated within very low, low, and medium vulnerability zones. This suggests that, under current hydrogeological conditions, the overall risk of widespread contamination remains relatively low. However, in settlements with inadequate wastewater handling—particularly those located in medium vulnerability zones—the potential for localized groundwater pollution cannot be entirely excluded, especially where the protective capacity of soil and subsurface layers may be limited by anthropogenic disturbance.

### 3.4 Validation of the Vulnerability Maps

The validation of the groundwater vulnerability map generated using the DRASTIC model is crucial for assessing the reliability of the spatial distribution of vulnerability zones. The aim of the validation is to determine whether the vulnerability classes identified on the map are capable of indicating the degree of protection against indicator parameters selected in parallel with dominant land use. In this study, the validation of the vulnerability map was performed by statistically evaluating the relationship between nitrate ion and Total Organic Carbon (TOC) concentrations—both of which represent major pollutant sources in the study area, namely domestic wastewater and agricultural activities. The correlation analysis was conducted using Pearson's correlation coefficient.

TOC concentration in groundwater has been recognized as a useful indicator of short residence time and, consequently, high intrinsic vulnerability, due to its rapid mineralization in the saturated zone.^[^ ^65^, ^66^ ^]^ Furthermore, because nitrate is naturally present in groundwater only at trace levels, its elevated concentrations are often attributed to anthropogenic inputs. Thus, nitrate is frequently used as a validation parameter in groundwater vulnerability assessments. Pollutant sources such as agricultural practices and domestic wastewater discharge can be identified by analyzing nitrate concentrations in groundwater.^[^ ^67^ ^]^

Several previous studies have utilized nitrate concentrations to validate DRASTIC‐based groundwater vulnerability models,^[^ ^40^, ^66^, ^68^, ^69^, ^70^, ^71^ ^]^ supporting its effectiveness as an indicator for model verification.

**Figure 12:**
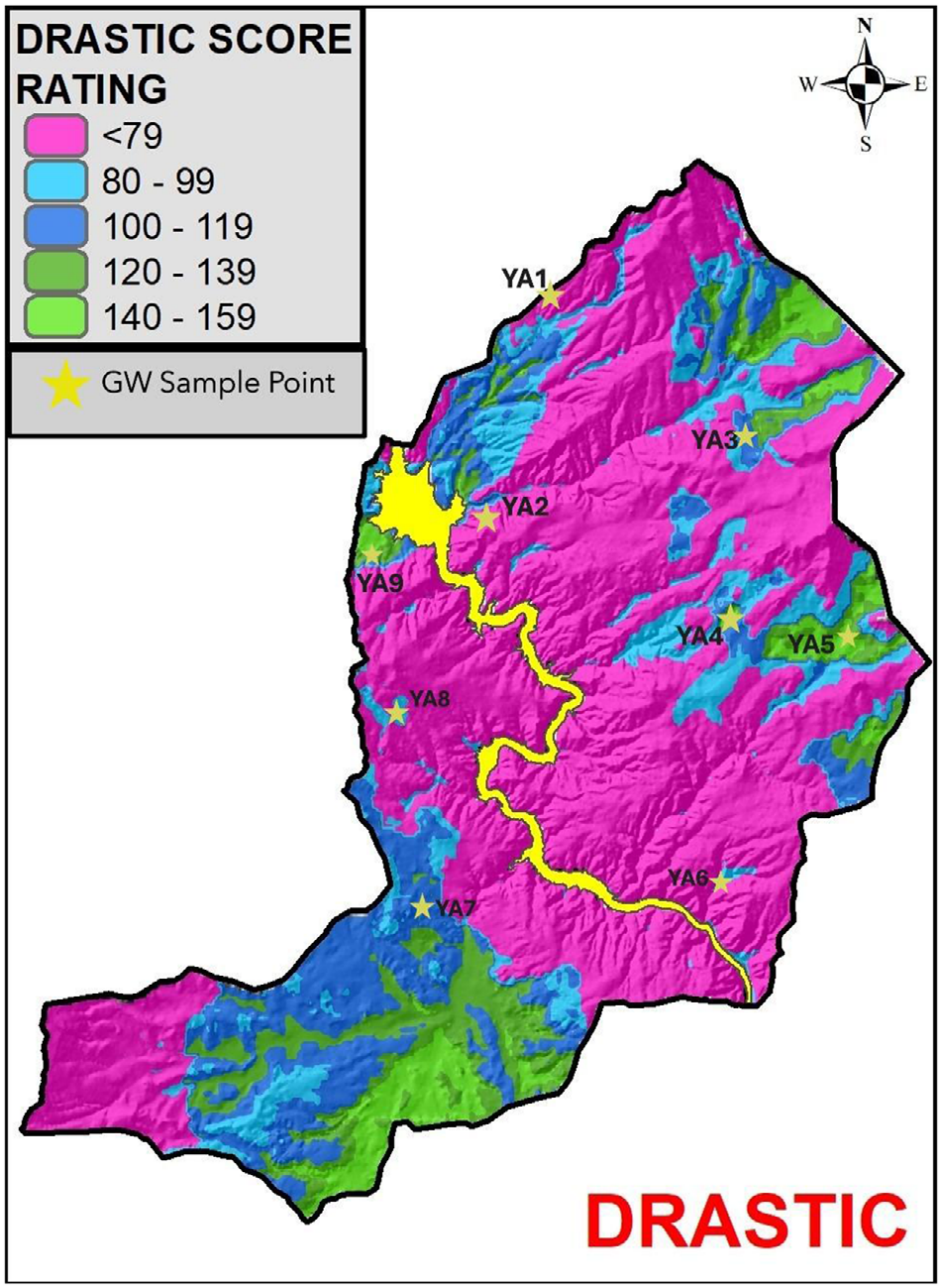
Groundwater sampling points overlaid on the groundwater vulnerability map.

Nitrate and TOC concentrations were analyzed in nine groundwater samples collected from the study area, and the results are provided in **Table** **13**. The table includes DRASTIC index scores corresponding to each sample location based on the map shown in **Figure** **12**.

**Table 13:** Kesikköprü Dam Lake Basin groundwater Nitrate, TOC, and DRASTIC index values.

GW sample point	Nitrate [mg L^−1^]	(TOC) [mg L^−1^]	DRASTIC INDEX
YA1	27	1.4	70
YA2	16	1.6	70
YA3	48	3.2	100
YA4	62	1.5	140
YA5	66	1.4	140
YA6	42	2	100
YA7	36	1.8	100
YA8	58	1.8	100
YA9	66	2.2	140

The relationship between the vulnerability DRASTIC index for the Kesikköprü dam lake basin and the concentrations of nitrate ions and TOC was estimated using the Pearson Correlation Coefficient (r). Pearson correlation is one of the most commonly used methods among correlation techniques, producing a score that can range from −1 to +1. Results close to +1 indicate a strong positive relationship between two variables, while results close to −1 indicate a strong negative (inverse) relationship between the two variables.^[^ ^72^ ^]^

The formula used to calculate the Pearson Correlation Coefficient (r) is provided below Equation (4)^[^ ^72^ ^]^

(4) $$r=\frac{n\left(\sum xy\right)-\left(\sum x\right)\left(\sum y\right)}{\sqrt{\left\{n\sum x^{2}\right\}}-{\left(\sum x\right)}^{2}\left\{\left\{n\sum y^{2}\right\}-{\left(\sum y\right)}^{2}\right\}}$$

r: Pearson correlation coefficient, indicates the degree and direction of a linear relationship between two variables.

n: The number of paired observations.

x: The values of the first variable (e.g., vulnerability index).

y: The values of the second variable (e.g., nitrate or TOC concentrations).

Recent studies have examined the association between TOC and nitrate in groundwater across various geographical contexts. For instance, Chandalsouk et al.^[^ ^73^ ^]^ reported a moderate positive correlation between TOC levels and groundwater vulnerability in Indonesia. In China, research showed that TOC and nitrate concentrations increased along groundwater flow paths and influenced microbial community structure.^[^ ^7^ ^]^ In Côte d'Ivoire, borehole water analysis revealed regional variations in TOC and nitrate levels, with some samples exceeding TOC standards.^[^ ^65^ ^]^ Ebeling et al.,^[^ ^74^ ^]^ found that agricultural land use strongly affects nitrate dynamics, while TOC concentrations were linked to riparian wetland density in German basins.

In the present study, a Pearson correlation analysis was performed using the data in Table 13 to assess the relationship between nitrate and TOC concentrations. The results revealed a strong positive correlation between the two parameters (*r* = 0.9995), consistent with previous studies.

This finding supports the conclusion that nitrate concentrations are reliable indicators for validating the vulnerability map. Furthermore, Pearson correlation analysis between nitrate concentrations and the DRASTIC index scores demonstrated a strong positive relationship (*r* = 0.9167). This indicates that nitrate concentrations were generally higher in areas classified as having higher vulnerability, and lower in zones of very low or low vulnerability. These results validate the consistency of the vulnerability classification developed for the Kesikköprü Dam Lake Basin and support the suitability of the DRASTIC model for groundwater contamination risk assessment in the study area.

## 4 Conclusions

This study provides a comprehensive assessment of groundwater vulnerability in the Kesikköprü Dam Lake Basin through the application of the GIS‐based DRASTIC index model. The outcomes highlight the spatial distribution of intrinsic vulnerability classes across the basin and offer critical insights for groundwater protection in a region supplying drinking water to Ankara.

The geological and hydrogeological analysis revealed that the basin is dominated by metamorphic and volcanic rock formations with inherently low permeability. The vadose zone is primarily composed of shale, which restricts vertical water movement. Soil classifications further corroborated the area's protective character, with a significant presence of clayey soils (Group D) contributing to low hydraulic conductivity and minimal recharge rates.

According to the DRASTIC model results, the majority of the basin falls under “very low” to “low” vulnerability zones, indicating limited susceptibility of groundwater to contamination under current natural conditions. However, “medium” vulnerability areas, though less extensive, are of particular concern due to their intersection with anthropogenic activities such as settlements with suboptimal wastewater management. These findings suggest that while overall risk remains low, localized vulnerabilities must be carefully managed.

Importantly, the DRASTIC model was supported through a statistical validation process using nitrate and TOC concentrations—key indicators of pollution pressure from agricultural and domestic sources. This validation affirms the reliability of the vulnerability map for practical planning purposes.

In conclusion, this study offers critical data for basin‐wide water resource planning, particularly for regulating future land use and mitigating pollution risks. The results emphasize the importance of preserving groundwater quality through proactive measures, strategic zoning, and targeted infrastructure investments. As pressures from climate change and population growth intensify, such integrative assessments will be vital for securing sustainable water resources in the region and beyond.

## Conflict of Interest

The authors declare no conflict of interest.

## Data Availability

The data that support the findings of this study are available from the corresponding author upon reasonable request.
